# Distal Femoral Dimensions in Turkish Population and Their Implications in Knee Prosthesis

**DOI:** 10.7759/cureus.75721

**Published:** 2024-12-14

**Authors:** Zennure Adiguzel Sahin, Ayse Derya Ertem, Elif Cansu Ibis, Sabri Kerem Diril, Hilmi Karadeniz

**Affiliations:** 1 Department of Anatomy, Istanbul University-Cerrahpasa, Cerrahpasa Medical Faculty, Istanbul, TUR; 2 Department of Orthopaedics and Traumatology, Bahcelievler State Hospital, Istanbul, TUR; 3 Department of Orthopaedics and Traumatology, Sportoteam Athlete and Spine Health Clinic, Istanbul, TUR

**Keywords:** bone, distal femur, femur anthropometry, total knee prosthesis, turkish population

## Abstract

Background: Total knee prosthesis is a frequently used material in surgery. Distal femur measurements must be taken into account to use the correct prosthesis. The aim of this study is to guide the development of a knee prosthesis suitable for distal femur dimensions in the Turkish population. Thus, it was planned to guide the Turkish population in choosing the most suitable knee prostheses imported from abroad.

Methods: Using 138 dry bones in the study, at first, the sides of the femurs were determined and eight parameters from femur distal and proximal parts were measured by a measuring tape and a digital caliper. Five parameters were measured at the distal part to evaluate whether these parameters differ according to the femur side.

Results: According to the results, distal femur measurements do not differ by side. Distal femur measurements in the Turkish population are wider than distal measurements of the Far East and Asian populations. These measurements are more similar to the measurements of the North American population. Also, the femur can be safely used in cases where there is no possibility of making a gender determination with the pelvis or the skull.

Conclusions: The femur's distinct morphological differences between genders offer invaluable insights for anthropometric gender determination. Our study underscores the importance of considering morphological and metric measurements and sets the stage for further research in this domain.

## Introduction

The femur is the longest and the most robust bone in the human body, forming the thigh skeleton. The distal end of the femur forms the knee joint in conjunction with the tibia and patella [1]. Over time, due to environmental, genetic, and mechanical factors, wear and tear and diseases can occur in the knee joint, which bears a significant portion of body weight. The most common disease observed is osteoarthritis. Osteoarthritis typically manifests itself with symptoms such as pain, swelling, and restricted movement [2].

Conservative and surgical treatment methods are applied to patients with osteoarthritis. For patients who do not respond to medical treatment, including arthroscopic approaches, knee arthroplasty surgery, a surgical treatment method, is performed [3,4]. The main principle in knee arthroplasties is to replace the damaged joint surface with prostheses made of metal on polyethylene. The replaced joint surface can be in the femur, tibia, or patella, or all three may need replacement [5]. There are numerous manufacturers designing knee prostheses. Using prostheses of appropriate size for patients is one of the most crucial factors affecting the success of the surgery and the patient's quality of life after the operation [6].

The femur is one of the bones with the highest probability of remaining intact. Femur measurement gives very successful results in gender determination. Therefore, it is frequently used in anthropological research and studies [7,8]. The dry bones used in this study belong to individuals living in Turkey. The data obtained are intended to assist in the use of appropriate imported knee prostheses for patients living in Turkey. This study attempted to determine the average anatomical measurements of the distal femoral end region of women and men living in Turkey. No comprehensive study has been conducted previously on distal femoral dimensions in the Turkish population. This study aims to guide the use of appropriately sized imported prostheses when needed.

The study was presented as an oral presentation at the 23rd National Anatomy Congress in 2023.

## Materials and methods

Permissions and ethical considerations

After obtaining institutional permissions from the centres where the measurements would be made, an application was made to the Clinical Research Ethics Committee of Istanbul University-Cerrahpaşa, Cerrahpaşa Medical Faculty. The study was initiated with permission number A-17, declared under number 7153, by our faculty's Ethics Committee convened on January 3, 2017.

Sample collection

A total of 138 dry femur samples were utilized in this study.

Measurement procedure

To minimize measurement error and maintain consistency of measurements, all measurements were taken by the same researcher on two consecutive days at the same time. At the end of the study, the reliability of the measurements was examined. The technical error of measurement and coefficient of reliability (R) methods were used to assess measurement reliability.

Measurement tools

Measurements were made using a measuring tape and a digital caliper with a precision of 0.01 mm.

Data collection and analysis

Initially, the femurs were numbered and all data obtained were recorded digitally. The femurs were oriented based on their heads for proximal ends and medial and lateral edges, and based on the intercondylar fossa for the anterior/posterior face. Subsequently, to determine gender, femur subtrochanter circumference (S1), caput femoris vertical diameter (S2), caput femoris transverse diameter (S3), caput femoris circumference (S4), height difference of femur epicondyles (S5), collum femoris sagittal diameter (S6), collum femoris transverse diameter (S7), and collum femoris length (S8) were measured [9].

**Figure 1:**
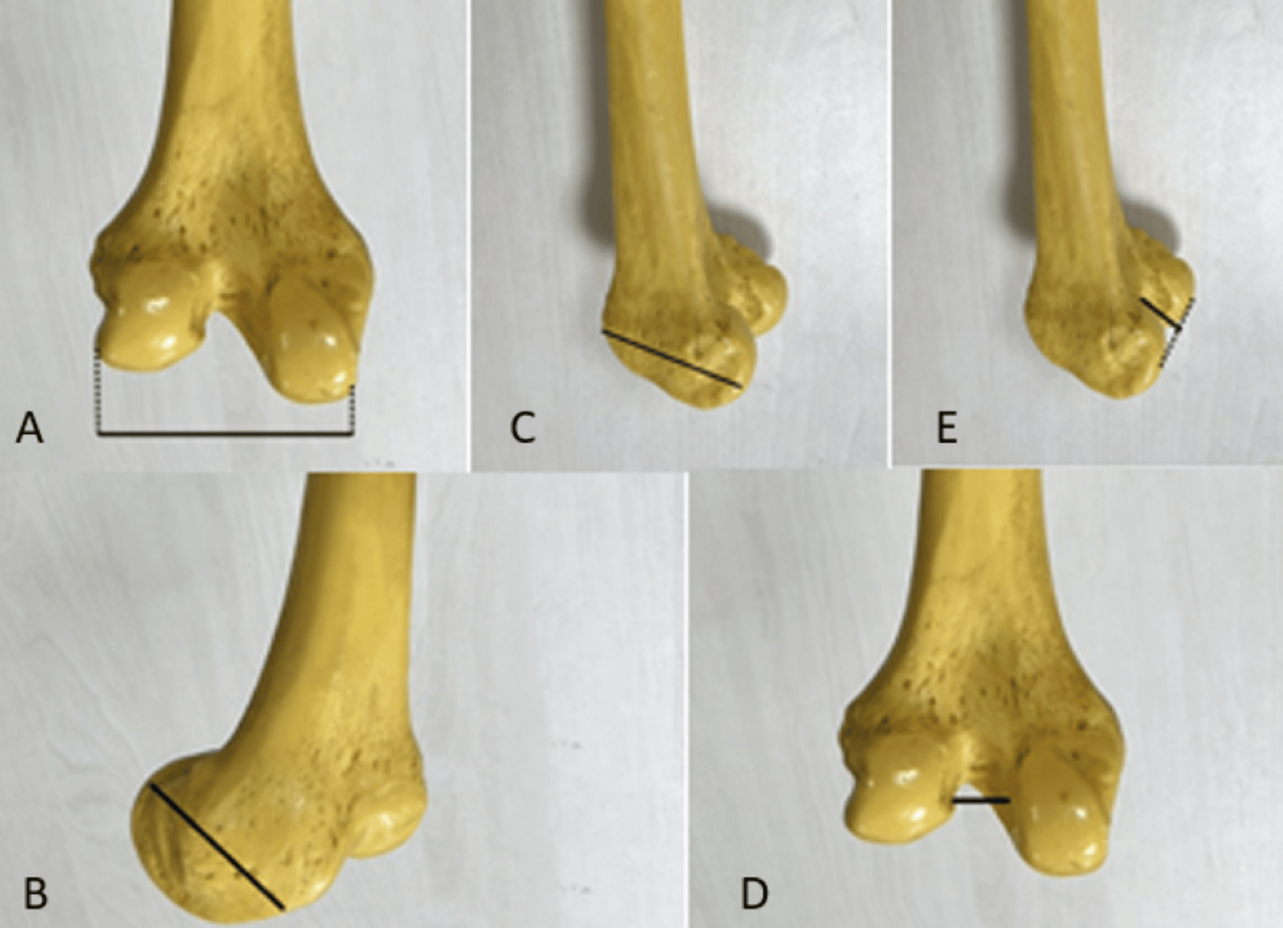
The measured femur parameters A: Bicondylar width (T1), B: condylus medialis depth (T2), C: condylus lateralis depth (T3), D: intercondylar width (T4), and E: intercondylus depth (T5).

After recording the measurement results to be used in gender determination, five different parameters were measured at the distal end of the femur, which was the main target of the study. The measured parameters are bicondylar width (T1), condylus medialis depth (T2), condylus lateralis depth (T3), intercondylar width (T4), and intercondylus depth (T5) (Figure 1).

Data analysis

The IBM SPSS Statistics 21.0 package program (IBM Corp, Armonk, NY) was used to evaluate the measurements. The McNemar test was used to compare the decisions made based on the receiver operating characteristic (ROC) curve's data and the measurements. In contrast, the Student's t-test was used to decide whether the femur distal end parameters changed according to direction and gender. A p-value of <0.05 was considered statistically significant.

## Results

The R-value for parameters S5 and S6 was less than 0.9, so they were excluded from the calculations, leaving six parameters. Therefore, at least four of the decision-making parameters evaluated for gender differentiation had to yield the same result (either male or female). One hundred and seventeen out of 138 bones met this condition.

The R-value for the variable T5 was less than 0.9, so it was excluded from the evaluation. Thus, the other four parameters measured at the distal end of the femur were evaluated.

Of the 117 bones evaluated, 63 were left-sided and 54 were right-sided. When gender differentiation was assessed using the 'S' values (also cross-checked with the ROC curve), it was determined that 59 bones belonged to females and 58 to males. The gender decisions made were statistically significant.

Statistical evaluation of the measurement results revealed significant differences in the averages of the 'T' variables between males and females (p<0.001). When considering whether there was a difference in femur distal end parameters by gender and side (right/left), significant differences were observed between males and females (p<0.05) for bicondylar width, medial condyle depth, and lateral condyle width (p=0.004 for width between medial and lateral condyles), but no significant difference was observed between right and left (p>0.05). According to this data, in the Turkish population, the average bicondylar width in the female femur distal end was 68.35 mm, while it was 75.23 mm in males; the depth of the medial condyle was 56.25 mm in females and 62.62 mm in males; the depth of the lateral condyle was 56.87 mm in females and 63.17 mm in males; the width between the condyles was 22 mm in females and 23.6 mm in males.

**Figure 2:**
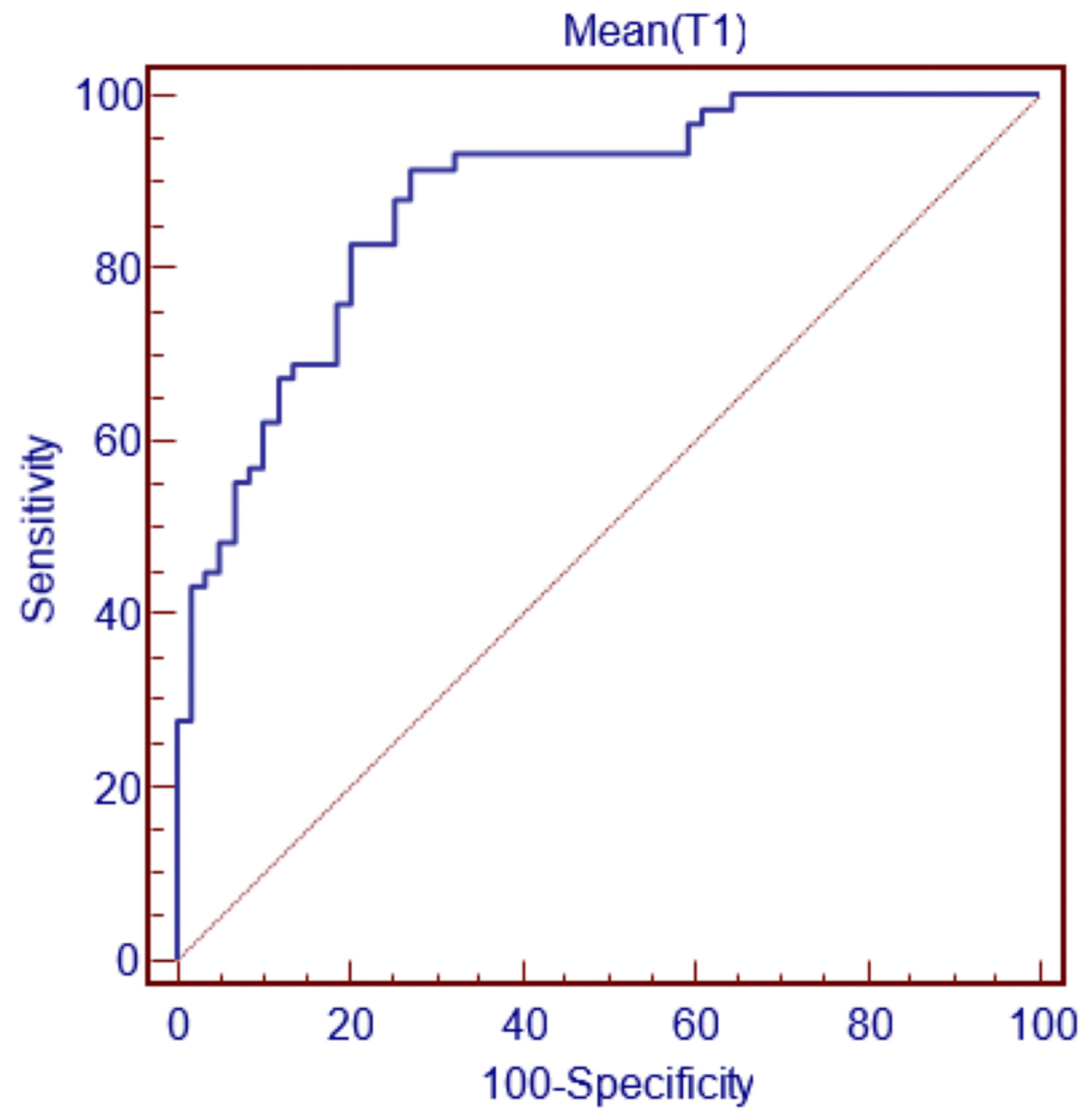
ROC curve for T1 ROC, receiver operating characteristic.

Additionally, in this study, decision-making ranges (estimation ranges) for some parameters used in gender differentiation were clarified using ROC curves. For gender decision-making with the bicondylar width parameter, if this parameter is less than 72.285 mm, the decision can be made as female, and if it is greater, as male, with 82.76% sensitivity and 79.66% specificity (Figure 2).

**Figure 3:**
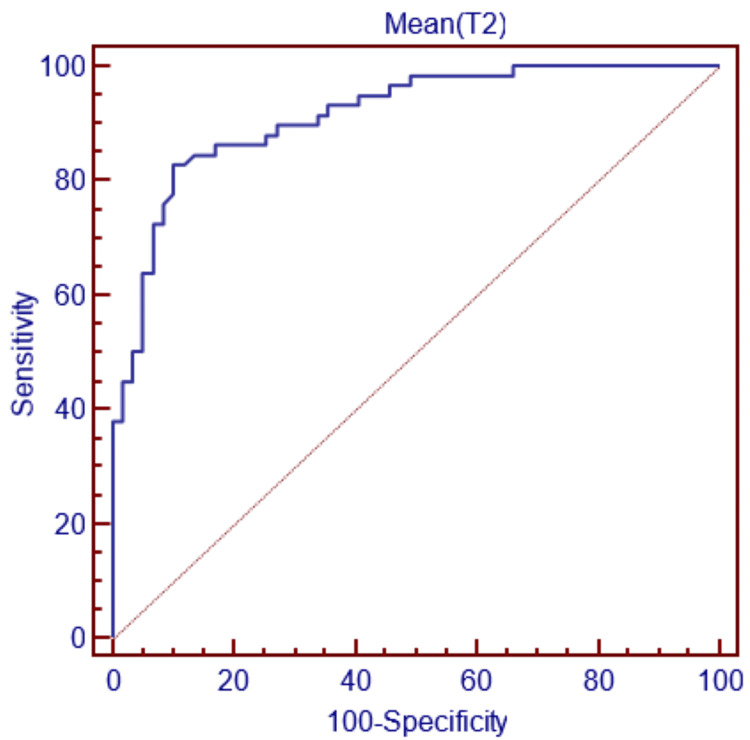
ROC curve for T2 ROC, receiver operating characteristic.

For gender decision-making with the condylus medialis depth parameter, if this parameter is less than 59.835 mm, the decision can be made as female, and if it is greater, as male, with 82.76% sensitivity and 89.83% specificity (Figure 3).

**Figure 4:**
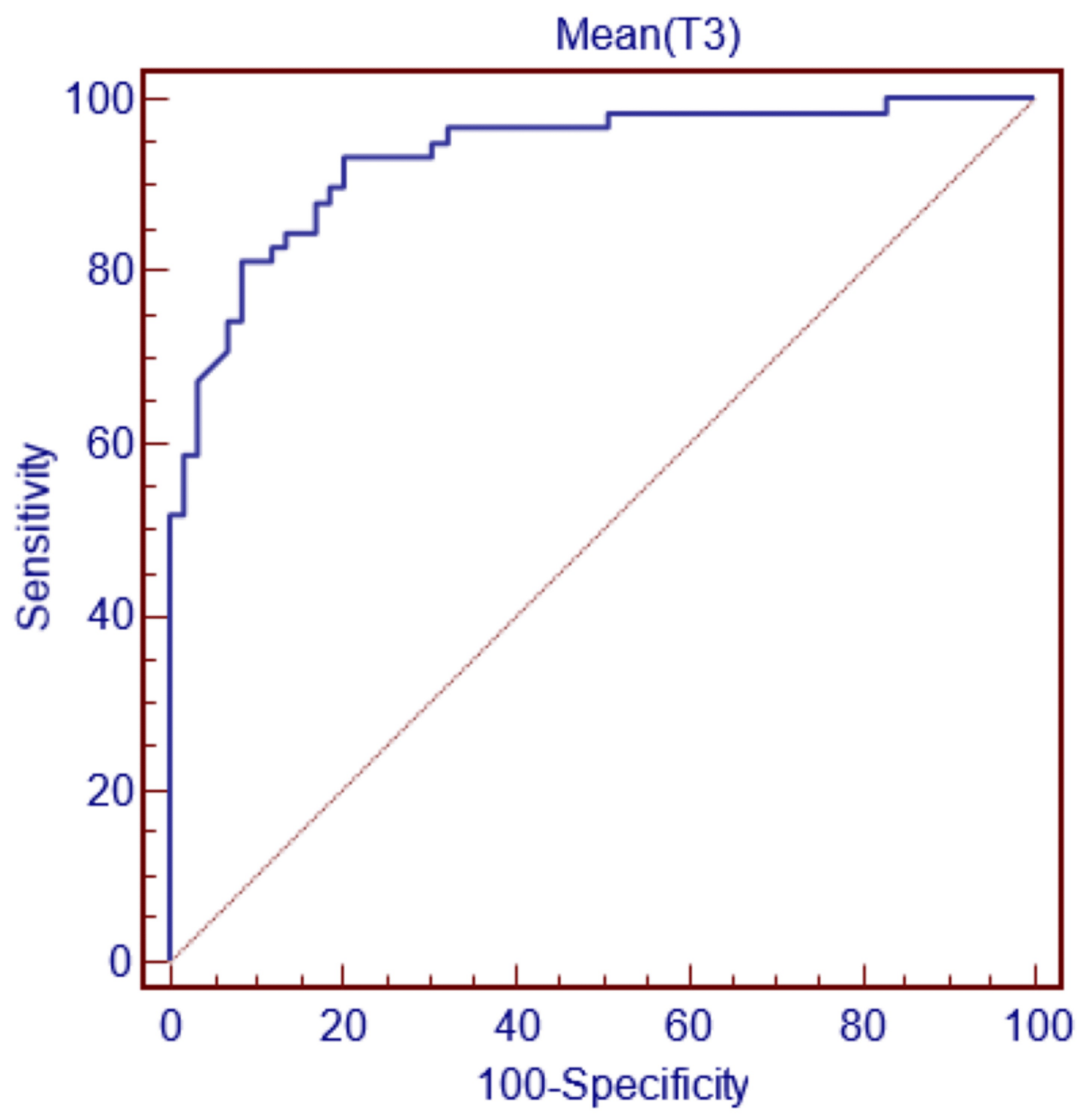
ROC curve for T3 ROC, receiver operating characteristic.

For gender decision-making with the condylus lateralis width parameter, if this parameter is less than 59.7 mm, the decision can be made as female, and if it is greater, as male, with 89.66% sensitivity and 81.36% specificity (Figure 4).

**Figure 5:**
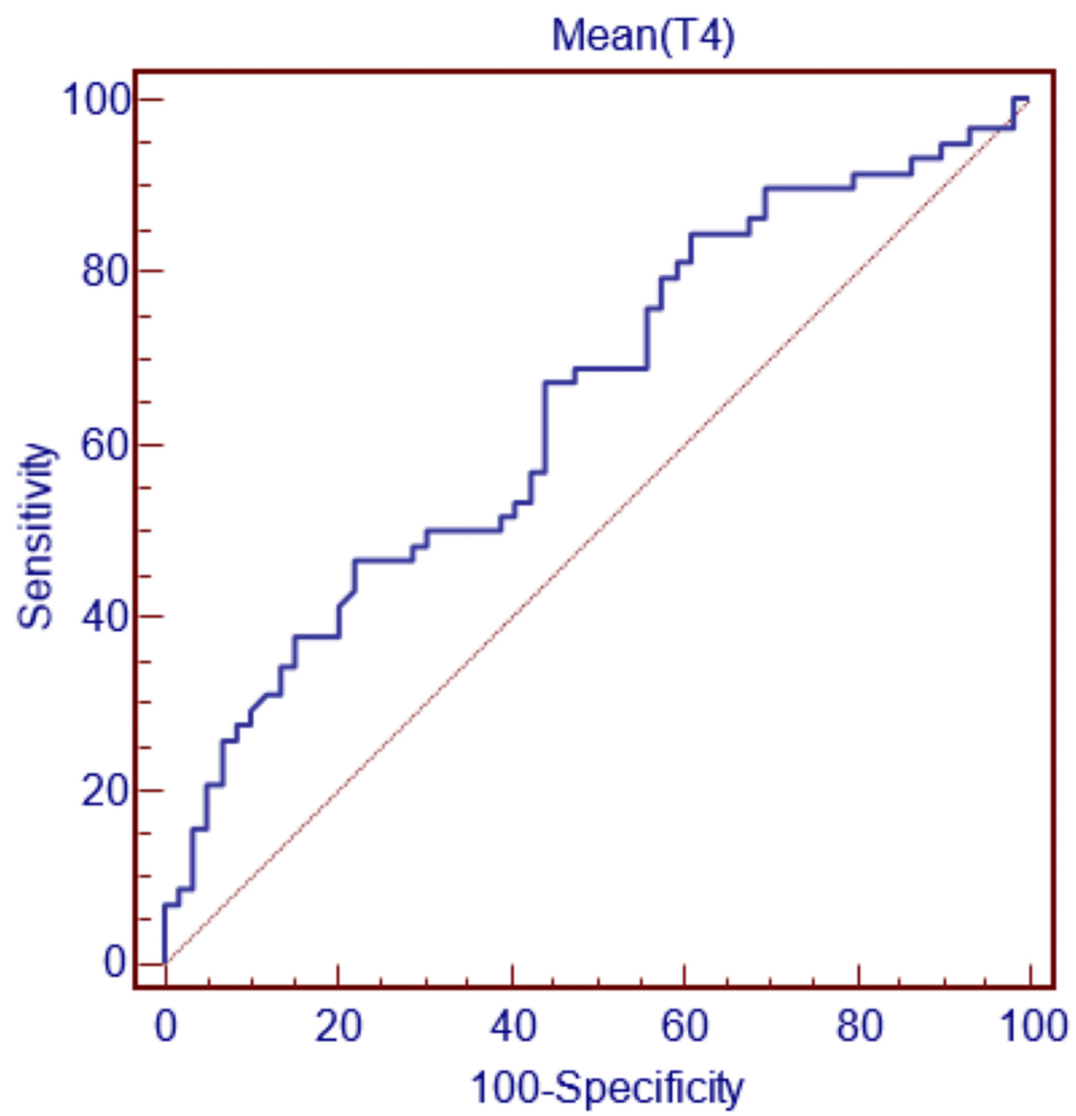
ROC curve for T4 ROC, receiver operating characteristic.

For gender decision-making with the width between medial and lateral condylus parameter, if this parameter is less than 23.02 mm, the decision can be made as female, and if it is greater, as male, with 53.45% sensitivity and 59.32% specificity (Figure 5; Table 1).

**Table 1:** Limit criteria used when determining gender with parameters measured at the distal end of the femur. Bicondylar width (T1), condylus medialis depth (T2), condylus lateralis depth (T3), and intercondylar width (T4). ROC analysis was used to determine the cut-off criteria. ROC, receiver operating characteristic.

Parameter	Our study			Kim and Friends' Study [10]		
	Female (mm)	Male (mm)	Accuracy (%)	Female (mm)	Male (mm)	Accuracy (%)
T1	<72.285	>72.285	82.76	<65.31	>65.31	90.1
T2	<59.835	>59.835	82.76	<58.67	>58.67	82.7
T3	<59.7	>59.7	89.66	<61.90	>61.90	81.7
T4	<23.02	>23.02	53.45	<20.52	>20.52	74.8

The graphs and table show that the width between medial and lateral condylus parameter does not have as high sensitivity and specificity in gender determination as the bicondylar width, condylus medialis depth, and condylus lateralis width parameters.

## Discussion

A comprehensive analysis of distal femur dimensions in the Turkish population is important in guiding the selection of imported prostheses to be applied. Bones in the human body, particularly the femur, can exhibit significant morphological differences between genders, making them invaluable tools in anthropometric studies for gender determination [11]. While the skull and pelvic bones are the primary choices for this purpose, in their absence or if they are in unusable conditions, long bones, especially the femur, are employed [12]. Both morphological and metric measurements are utilized for gender determination using the femur [13]. In our study, even though we predominantly used the proximal end of the femur for gender determination, the results suggest that the parameters bicondylar width (T1), condylus medialis depth (T2), and condylus lateralis (T3) width can be confidently employed for this purpose. The potential of these parameters in gender determination has not been previously described. If future researchers validate and find these parameters reliable, they could be introduced into the literature as definitive gender-determining parameters.

Beyond its significance in gender determination, the femur's anatomy needs thorough understanding, especially considering the rising global incidence of knee problems and the subsequent total knee arthroplasties [14]. The primary objective of knee arthroplasty is to alleviate pain and enhance function [15]. To achieve this, it is imperative to employ the correct surgical method and an appropriately sized prosthesis [15]. A well-fitted prosthesis should possess the right shape and size [16]. There can be variations in the distal end measurements of the femur between races and genders. Given that some prostheses in Turkey are imported, understanding these variations is crucial, ensuring that knee prostheses are procured with these racial and gender differences in mind.

Recent studies have aimed to determine the differences in the femur based on gender and race, with varying results. Our analysis and others conducted in China, Korea, America, and Thailand consistently found that female distal femur measurements are narrower than males. Specifically, in our study, the femoral bicondylar width, medial and lateral condyle depths, and intercondylar width and depth were narrower in females. This finding is consistent with the study on 220 digital Korean femur models [10]. Yue et al.'s examination of 40 Chinese femurs [17] and Wu et al.'s research on 141 dry bones in the Chinese population [18] all showed significant gender differences in these parameters. Mensch and Amstutz's evaluation of North American X-rays and cadaver femurs [19] and Chaichankul's study using 200 MRI scans in the Thai population [10] also reported similar gender disparities. However, Pinskerova's study in Prague found no statistically significant gender differences in three of four femoral measurement parameters [20].

Comparative studies in different populations reveal exciting insights. Vaidya et al.'s study of the Indian population using 86 knee CT scans [21] and another study of knee MRIs in 535 Asian patients [22] showed that Asian femur measurements were generally narrower than those in Western populations. The dimensions of the distal femur in our study were wider than in the Indian population, but narrower than Western standards, especially in the medial and lateral condyle depths. In addition, another study from Germany also highlighted differences in medial and lateral condyle measurements between genders [23]. These studies collectively suggest that while there are consistent gender differences in femur measurements, racial and regional variations also play a significant role, underscoring the importance of considering these factors in medical and anthropological research.

Limitations of the study

One of the main limitations of our study is its reliance on dry bones. Dry bones are often not the first choice for research due to several challenges. First, the availability of a sufficient number of specimens can be a significant constraint, limiting the sample size and potentially the generalizability of the results. Second, dry bones may not always be representative of the wider population, as they may be from specific subgroups or time periods, introducing potential bias.

Furthermore, measurements on dry bone can sometimes be more challenging than on live tissue or radiological images. The absence of soft tissue can make certain landmarks less visible, potentially introducing measurement errors. Dry bones may also have undergone postmortem changes or damage, further complicating measurements.

Another limitation is the potential lack of comparability between our results and studies using other methods, such as radiological imaging. Although we aimed to fill this gap by comparing our results with those from radiological studies, there may still be inherent differences between the two methods.

Finally, although our study provides valuable insights into femur-based sex determination, it is important to note that there may be individual variations and our findings may not apply universally to all populations or ethnic groups.

## Conclusions

The femur's distinct morphological differences between genders offer insights into using the femur for gender determination. Racial variability should be taken into account for the dimensions of domestically produced and imported knee prostheses. Our study underscores the importance of considering morphological and metric measurements and sets the stage for further research in this domain.
